# Diabetic Bone Marrow Cell Injection Accelerated Acute Pancreatitis Progression

**DOI:** 10.1155/2021/5123823

**Published:** 2021-08-21

**Authors:** Xiao-Min Luo, Cen Yan, Yue-Jie Zhang, Ling-Jia Meng, Guo-Tao Lu, Ji-Ming Yin, Ying-Mei Feng

**Affiliations:** ^1^Department of Science and Technology, Beijing Youan Hospital, Capital Medical University, Beijing 100069, China; ^2^Pancreatic Center, Department of Gastroenterology, Affiliated Hospital of Yangzhou University, Yangzhou University, Yangzhou 225099, China; ^3^Beijing Hepatology Institute, Beijing 100069, China

## Abstract

Acute pancreatitis (AP) is one of the leading causes of hospital admission, 20% of which could progress to the severe type with extensive acinar cell necrosis. Clinical studies have reported that diabetes is an independent risk factor of the incidence of AP and is associated with higher severity than nondiabetic subjects. However, how diabetes participates in AP progression is not well defined. To investigate this question, wild-type (wt) and diabetic db/db mice at the age of 16 weeks were used in the study. AP was induced in wt recipients by 10 injections of 50 *μ*g/kg caerulein with a 1 h interval. One hour after the last caerulein injection, bone marrow cells (BMC) isolated from wt and db/db mice were injected intraperitoneally into the recipients (1 × 10^7^cells/recipient). The recipients with no BMC injection served as controls. Thirteen hours after BMC injection, serum lipase activity was 1.8- and 1.3-folds higher in mice that received db/db BMC, compared with those with no injection and wt BMC injection, respectively (*p* ≤ 0.02 for both). By H&E staining, the overall severity score was 14.7 for no cell injection and 16.6 for wt BMC injection and increased to 22.6 for db/db BMC injection (*p* ≤ 0.002 for both). In particular, mice with db/db BMC injection developed more acinar cell necrosis and vacuolization than the other groups (*p* ≤ 0.03 for both). When sections were stained with an antibody against myeloperoxidase (MPO), the density of MPO+ cells in pancreatitis was 1.9- and 1.6-folds higher than wt BMC and no BMC injection groups, separately (*p* ≤ 0.02 for both). Quantified by ELISA, db/db BMC produced more IL-6, GM-CSF, and IL-10 compared with wt BMC (*p* ≤ 0.04 for all). In conclusion, BMC of db/db mice produced more inflammatory cytokines. In response to acinar cell injury, diabetic BMC aggravated the inflammation cascade and acinar cell injury, leading to the progression of acute pancreatitis.

## 1. Introduction

Diabetes is a common disease worldwide. According to the recent report, the number of diabetic patients was estimated at 415 million in 2015 and might be projected to be 642 million by 2040 [1]. Type 2 diabetes mellitus (T2DM) accounted for more than 90% among diabetic patients, which is featured as insulin resistance in peripheral organs and insufficient insulin production by dysfunctional pancreatic *β* cells [1]. In a meta-analysis involving 698,782 T2DM patients, the hazard ratio was 2.00 (95% CI, 1.83-2.19) for coronary heart disease, 2.27 (95% CI, 1.95-2.65) for ischemic stroke, and 1.73 (95% CI, 1.51-1.98) for other vascular diseases, respectively, compared with nondiabetic subjects [2].

Apart from vascular complications described above, acute pancreatitis (AP) occurs more frequently in diabetic patients than nondiabetic ones [3, 4]. In a prospective study (*n* = 547,554), the adjusted hazard ratio of having acute pancreatitis was 1.53 (95% CI, 1.49-1.58) for diabetic patients after 8 years of follow-up. Moreover, the presence of diabetes was associated with a 1.46-fold increased risk of severe AP compared with nondiabetic ones [3]. Likewise, a meta-analysis of 354,880 patients revealed that the risk of having AP was 1.55 (95% CI, 1.27-1.90) for diabetic patients compared with nondiabetic subjects. Furthermore, the risk of getting intensive care admission and organ failure was 1.80- and 1.59-folds greater in diabetic patients than nondiabetic individuals [4]. In line with clinical findings, when challenged with caerulein injection, diabetic db/db mice developed more severe AP than wt controls [5]. Nevertheless, how diabetes potentiates AP progression is not fully known.

As a sort of aseptic inflammation, AP is manifested as inflammatory cell infiltration and acinar cell necrobiosis. In the early stage of AP, acinar cells produce tumor necrosis factor- (TNF-) *α*, IL-10, and monocyte chemoattractant protein-1 (MCP-1) which promote neutrophil and subsequent monocyte infiltration into the panaceas. These inflammatory cells enhance inflammatory cytokine production such as IL-6 and TNF-*α* and produce enzymes, both of which orchestrate acinar cell autoaggression and destruction [6, 7]. In type 2 diabetic patients, the ratio of neutrophils versus lymphocytes was higher than that of nondiabetic participants and subjects with impaired glucose tolerance [8]. Given that circulating white blood cells originated from bone marrow cells (BMC), we hypothesized that BMC from diabetic mice could contribute to AP progression than nondiabetic BMC. To investigate this question, wt mice received caerulein injections to induce AP. One hour after the last caerulein injection, BMC of C57BL/6 and age-matched diabetic db/db mice were isolated and injected intraperitoneally into the recipients to assess disease severity.

## 2. Method

### 2.1. Animals

Male C57BL/6 and db/db mice with a C57BL/6 background at the age of 8 weeks were purchased from Beijing Vital River Laboratory Animal Technology Co., Ltd. and GemPharmatech Co., Ltd., respectively. They were housed under specific pathogen-free (SPF) conditions in an air-conditioned animal facility at 24°C on a twelve-hour light/dark cycle. Animals had free access to water and standard laboratory chow ad libitum. The study protocol was approved by the experimental animal ethics committee of Capital Medical University.

As reported, db/db mice developed diabetes at the age of 14-16 weeks [9, 10]. Therefore, lipid and glucose levels, COFO data, and BMC isolation were performed in db/db mice and wt controls at the age of 16 weeks.

### 2.2. Mouse Model of AP

The murine model of AP could be induced by a series of intraperitoneal injections of caerulein [5, 11, 12]. After being fasted overnight, C57BL/6 mice at the age of 8 weeks received intraperitoneal injection of 50 *μ*g/kg caerulein. A total of 10 injections with a one-hour interval were performed to establish AP. To obtain consistent induction of AP, a fresh solution of caerulein at the final concentration was prepared prior to each experiment (Cae, AnaSpec, Inc., San Jose, CA, USA).

### 2.3. BMC Isolation and Cell Injection Experiment

BMC were isolated from C57BL/6 and db/db mice at the age of 16 weeks. Briefly, tibias and femurs were dissected free. After making a small cut on one edge of the bone, the bone cavity was flushed with 5 mL sterile PBS. After, BMC were spun down by centrifugation at 700 g for 5 min. One hour after the 10^th^ caerulein injection of caerulein, BMC were administered intraperitoneally to the recipients with induced AP (1 × 10^7^ cells/recipient). The recipients were randomly assigned to 3 groups: no BMC injection (*n* = 6), C57BL/6 BMC injection (*n* = 15), and db/db BMC injection (*n* = 15). In parallel, the mice injected with saline alone served as pancreatitis-free controls.

Blood samples were obtained from the tail vein of the mice at 0, 12, and 24 hours before and after the first caerulein injection. The animals were anesthetized by intraperitoneal injection of sodium pentobarbital (50 mg/kg) and sacrificed 24 hours after the first caerulein injection. The pancreatic tissues were dissected, fixed in 4% paraformaldehyde in PBS, and embedded in paraffin for histological analysis.

### 2.4. ELISA

Plasma levels of total cholesterol and triglyceride were determined according to the protocols (Nanjing Jiancheng Corp., Nanjing, China). Fasting blood glucose levels were measured based on the manufacturer's instruction (Roche). Plasma insulin levels were measured by ELISA (Cat. No. CEA448Mu, Cloud-Clone Corp., Wuhan, China). Plasma lipase and amylase activities were measured by commercial kits and quantified by a Tecan Safire microplate reader (mouse amylase assay kit: ab102523, Abcam, Cambridge, MA; lipase kits: Nanjing Jiancheng Corp., Nanjing, China).

### 2.5. Cytokine Measurement

Proteins were extracted from BMC of wt and db/db mice using the RIPA buffer (MILLIPLEX® MAP, USA). Protein concentration was quantified by the BCA method (Thermo Scientific, USA). After calculation, 25 *μ*g proteins of each sample were loaded to a 96-well plate and mixed with mouse high-sensitivity T cell antibody-immobilized premixed magnetic beads. A panel of cytokines was determined by MILLIPLEX® MAP following the manual instruction. The cytokines included granulocyte-macrophage colony-stimulating factor (GM-CSF), interferon-gamma (IFN-*γ*), interleukin-1alpha (IL-1*α*), interleukin-1beta (IL-1*β*), interleukin-2 (IL-2), interleukin-4 (IL-4), interleukin-5 (IL-5), interleukin-6 (IL-6), interleukin-7 (IL-7), interleukin-10 (IL-10), interleukin-12 (IL-12) (p70), interleukin-13 (IL-13), interleukin-17A (IL-17A), keratinocyte chemokine (KC), lipopolysaccharide-induced CXC chemokine (LIX), MCP-1, macrophage inflammatory protein-2 (MIP-2), and TNF-*α*.

### 2.6. Histology

The paraffin-embedded pancreatic tissue was cut into sections with a 4 *μ*m thickness. After hematoxylin and eosin staining, the severity in the aspects of inflammation, acinar cell necrosis and vacuolization, and edema of the pancreas was scored to compute the total severity. Pathological changes were assessed by two investigators blinded to the experiments. At least six visual fields of each mouse were studied.

### 2.7. Immunohistochemistry

Paraffin-embedded sections were deparaffinized and incubated with a 0.3% H_2_O_2_ solution to remove endogenous peroxidase activity. Antigen retrieval was performed with a citrate buffer (pH 6.8). Goat serum was used to block nonspecific antigens. Then, paraffin sections were probed with a rabbit anti-mouse myeloperoxidase (MPO) polyclonal antibody (dilution: 1/1000; GB11224, Servicebio, Wuhan, China) or with an anti-insulin mouse monoclonal antibody (dilution: 1/500; GB13121, Servicebio, Wuhan, China). The next day, sections were incubated with a goat anti-rabbit secondary antibody or goat anti-mouse secondary antibody for 1 hour at room temperature. A diaminobenzene horseradish peroxidase color development kit was used for the color reaction. Finally, the sections were counterstained with hematoxylin and dehydrated. Sections stained only with the secondary antibody with the omission of the primary antibody served as negative controls. The staining-positive cells in the pancreas were counted using the ImageJ software. At least six visual fields of each mouse were studied. Data were expressed as MPO+ cells per microscopy field and the percentage of insulin+ cells in islets [13].

### 2.8. Statistical Analysis

Data were presented as mean ± SD. An unpaired, 2-tailed Student's test was applied to compare the means. One-way analysis of variance (ANOVA) with Dunnett's multiple comparison test was applied when there were more than two experimental groups. Significance was a two-tailed *p* value of 0.05 or less.

## 3. Results

### 3.1. Diabetic db/db Mice Were Featured as Myeloid Cell Expansion

**Figure 1:**
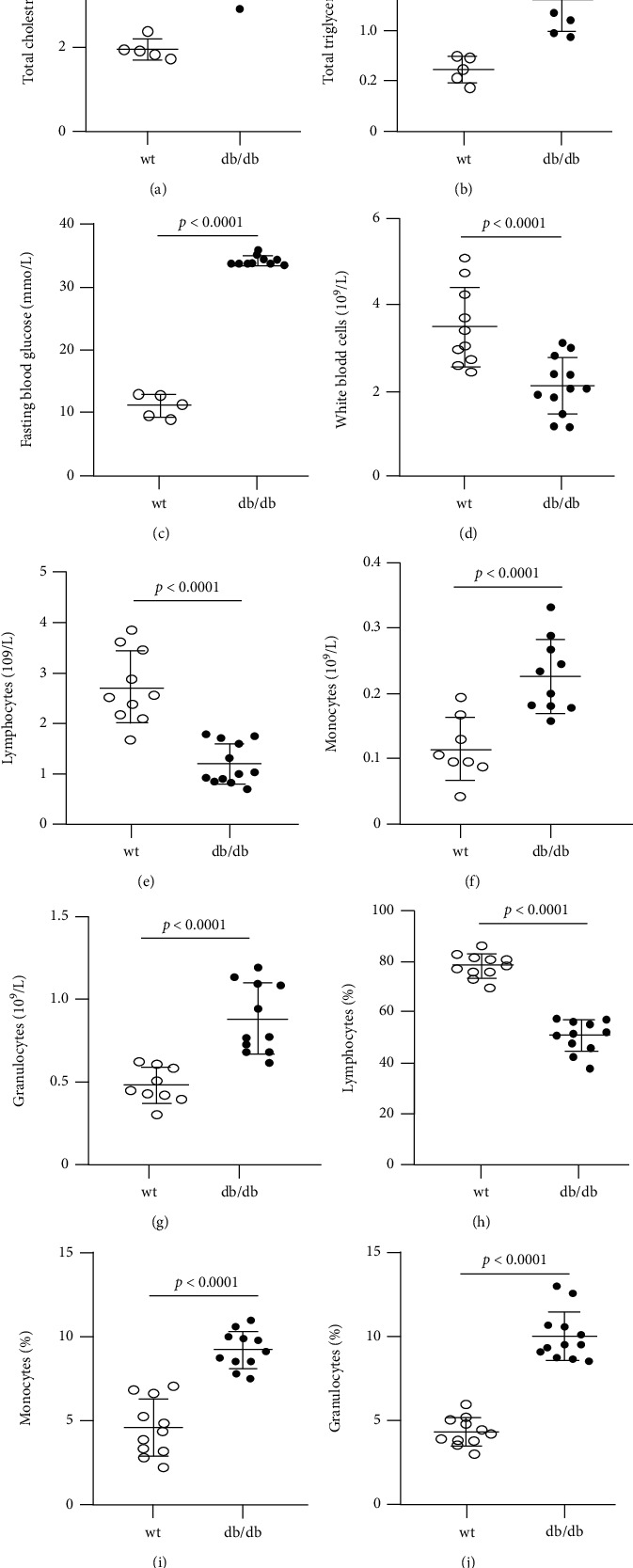
Phenotypic characteristics of wt and db/db mice at the age of 16 weeks. (a) Fasting plasma cholesterol, *n* = 5-10. (b) Fasting plasma triglyceride, *n* = 5-10. (c) Fasting blood glucose, *n* = 10-12. (d–g) White blood cell count and differential white blood cell count. (h–j) Proportion of lymphocytes, monocytes, and granulocytes among white blood cells, *n* = 10-12. wt: wild type.

At the age of 16 weeks, plasma levels of cholesterol and triglyceride were 2.2- and 2.5-folds higher in db/db mice than wt controls (Figures 1(a) and 1(b)). Hyperglycemia started to appear in obese db/db mice at the age of 8 weeks and to progress following the age. By the age of 14-16 weeks, *β* cell content was decreased in islets with a reduced *β* cell mass and impaired insulin secretion, indicating *β* cell dysfunction [9, 10]. Similar to these reports, fasting blood glucose levels were 11.02 mmol/L at wt mice but increased to 34.11 mmol/L at db/db mice (Figure 1(c)).

Compared with wt mice at 16 weeks old, the number of white blood cells and lymphocytes in the peripheral blood was 40% and 56% lower in db/db mice (white blood cells: 3.48 ± 0.92 × 10^9^/L vs. 2.10 ± 0.66 × 10^9^/L, *p* = 0.01, and *n* = 11-13; lymphocytes: 2.58 ± 0.76 × 10^9^/L vs. 1.32 ± 0.53 × 10^9^/L, *p* = 0.0002, and *n* = 11-13) (Figures 1(d) and 1(e)). The number of monocytes was comparable between two groups (monocytes: 0.16 ± 0.08 × 10^9^/L vs. 0.19 ± 0.08 × 10^9^/L, *p* = 0.33, and *n* = 11-13) (Figure 1(f)). However, granulocyte and monocyte counts were 1.8- and 2.0-folds higher in db/db mice, respectively, compared with wt controls (granulocytes: 0.48 ± 0.11 × 10^9^/L vs. 0.88 ± 0.21 × 10^9^/L, *p* < 0.0001, and *n* = 11-13; monocytes: 0.11 ± 0.05 × 10^9^/L vs. 0.23 ± 0.06 × 10^9^/L, *p* = 0.0004, and *n* = 11-13) (Figure 1(g)). Accordingly, the proportion of lymphocytes was 35% lower, but the percentage of monocytes and granulocytes was 2.0- and 2.3-folds higher in db/db mice, respectively, when compared with wt controls (lymphocytes: 78.1 ± 4.7% vs. 50.7 ± 6.2%, *p* < 0.0001; monocytes: 4.6 ± 1.7% vs. 9.3 ± 1.1%, *p* < 0.0001; and granulocytes: 17.3 ± 3.4% vs. 40.2 ± 5.9%, *p* < 0.0001) (Figures 1(h)–1(j)).

General characterization of age-matched male wt and db/db mice is summarized in Table 1.

### 3.2. The Effect of BMC Injection on Serum Amyloid and Lipase Activity in Mice with Induced Acute Pancreatitis

**Figure 2:**
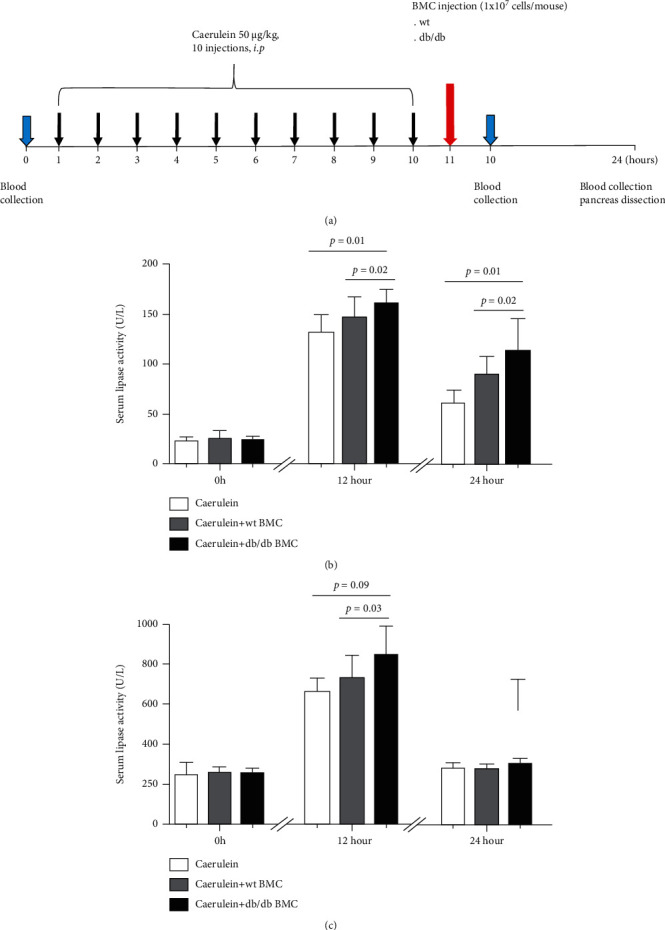
Biochemical analysis of AP in mice that received bone marrow cell (BMC) injection. (a) The scheme of the study design. (b, c) Serum lipase activity and serum amylase activity in mice with induced AP following BMC injection. AP was induced in wt recipients by ten caerulein injections. One hour after the last injection, BMC of wt or db/db donors were isolated and injected into mice with induced AP. AP: acute pancreatitis; wt: wild type; BMC: bone marrow cells.

Previously, Gao et al. reported that db/db mice developed severe acinar damage than wt controls after caerulein injection [5]. To testify whether BMC contributed to acute pancreatitis progression, BMC were isolated from wt and db/db donors and injected peritoneally into the caerulein-injected mice. The recipients without BMC injection served as controls. The scheme of the experimental design is illustrated in Figure 2(a).

Quantified by ELISA, serum lipase activity was comparable prior to injection among three groups (no injection: 23.5 ± 3.8 U/L, *n* = 3; wt BMC: 25.4 ± 8.0 U/L, *n* = 15; and db/db BMC: 24.4 ± 4.5 U/L, *n* = 15; *p* ≥ 0.57) (Figure 2(b)). Following the first injection, serum lipase levels were dramatically increased. One hour after BMC injection, serum lipase levels were increased to 132.5 U/L for the no injection group, 145.1 U/L for the wt BMC group, and 161.1 U/L for the db/db BMC group (132.5 ± 17.1 U/L vs. 145.1 ± 21.0 U/L vs. 161.1 ± 14.8 U/L, *n* = 6-15). Similar findings were observed at 13 hours after BMC injection (serum lipase levels: 62.0 ± 13.3 U/L vs. 89.6 ± 18.6 U/L vs. 113.6 ± 32.6 U/L) (Figure 2(b)).

Similar kinetics of serum amylase was detected. Compared with no BMC injection, serum amylase activity was 1.3- and 1.1-folds higher after 1 and 13 hours of db/db BMC injection (1 h: 663.3 ± 67.3 U/L vs. 845.8 ± 147.0 U/L, *n* = 6-15, and *p* = 0.009; 13 h: 279.7 ± 26.8 U/L vs. 302.7 ± 22.2 U/L, *n* = 6-15, and *p* = 0.03). By contrast, serum amylase activity was not altered between the wt BMC injection and no injection groups in the entire experiment (1 h after BMC injection: 663.3 ± 67.3 U/L vs. 735.3 ± 111.0 U/L, *n* = 6-15, and *p* = 0.16; 13 h after BMC injection: 279.7 ± 26.8 U/L vs. 282.5 ± 17.7 U/L, *n* = 6-15, and *p* = 0.78) (Figure 2(c)).

### 3.3. The Effect of BMC Injection on Histological Changes in Mice with Induced Acute Pancreatitis

**Figure 3:**
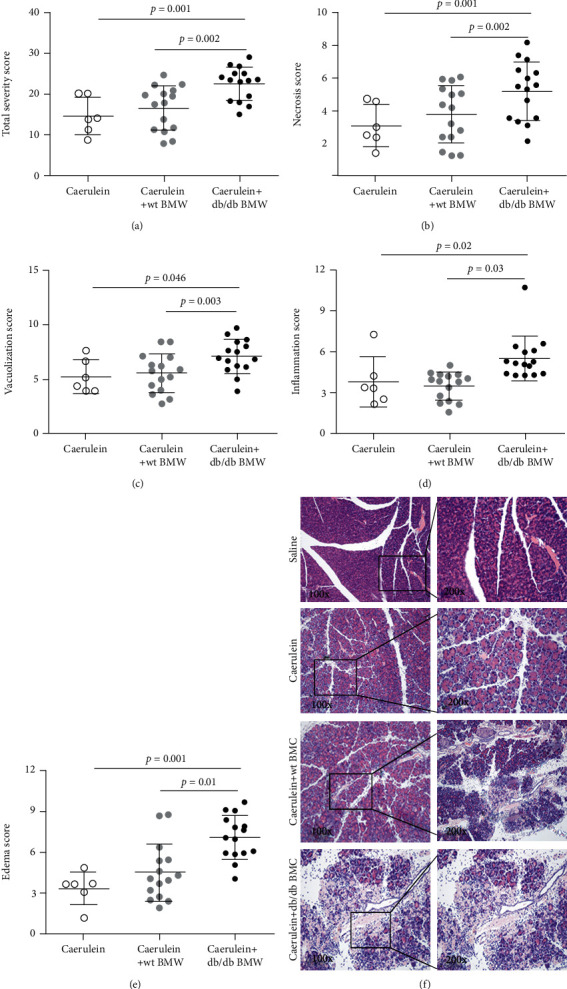
The effect of bone marrow cell injection on AP progression. The severity was assessed on H&E-stained sections in the aspects of acinar cell necrosis and vacuolization, inflammatory cell infiltration, and edema of the pancreas. The degree of severity was scored from 1 to 10. Total severity score (a) was the sum of necrosis (b), vacuolization (c), inflammation (d), and edema (e). *n* = 6-15. AP: acute pancreatitis.

As previously described [5, 14, 15], the pancreatitis damage was assessed in the aspects of inflammation as evidenced by infiltrated inflammatory cells, acinar cell vacuolization and necrosis, and edema in the pancreas. The overall total severity score was 14.7 for no injection, 16.6 for wt BMC injection, and 22.6 for db/db BMC injection (*p* = 0.001 for db/db BMC injection vs. no injection; *p* = 0.002 for db/db BMC vs. wt BMC) (Figure 3(a)). Compared with the normal pancreas, the extent of pancreas injury induced by caerulein injection alone or with wt BMC injection was similar, both of which were presented by a low degree of edema and a small amount of infiltrated inflammatory cells and necrotic acinar cells. Compared with other groups, db/db BMC injection induced pronounced acinar cell necrosis and vacuolization, indicating the severe damage in situ (necrosis score: 3.1 ± 1.3 vs. 3.8 ± 1.8 vs. 5.2 ± 1.8, *n* = 6-15; vacuolization score: 3.8 ± 1.8 vs. 3.5 ± 1.0 vs. 5.5 ± 1.6; *n* = 6-15) (Figures 3(b) and 3(c)). Accordingly, more severe inflammation and edema were observed in mice that received db/db BMC injection in comparison to the other groups (edema score: 3.3 ± 1.2 vs. 4.5 ± 2.1 vs. 7.1 ± 1.6; inflammation score: 5.3 ± 1.6 vs. 5.5 ± 1.8 vs. 7.1 ± 1.6; *n* = 6-15) (Figures 3(d) and 3(e)). The representative H&E sections are shown in Figure 3(f).

### 3.4. The Effect of BMC Injection on Neutrophil Infiltration in Mice with Induced Acute Pancreatitis

**Figure 4:**
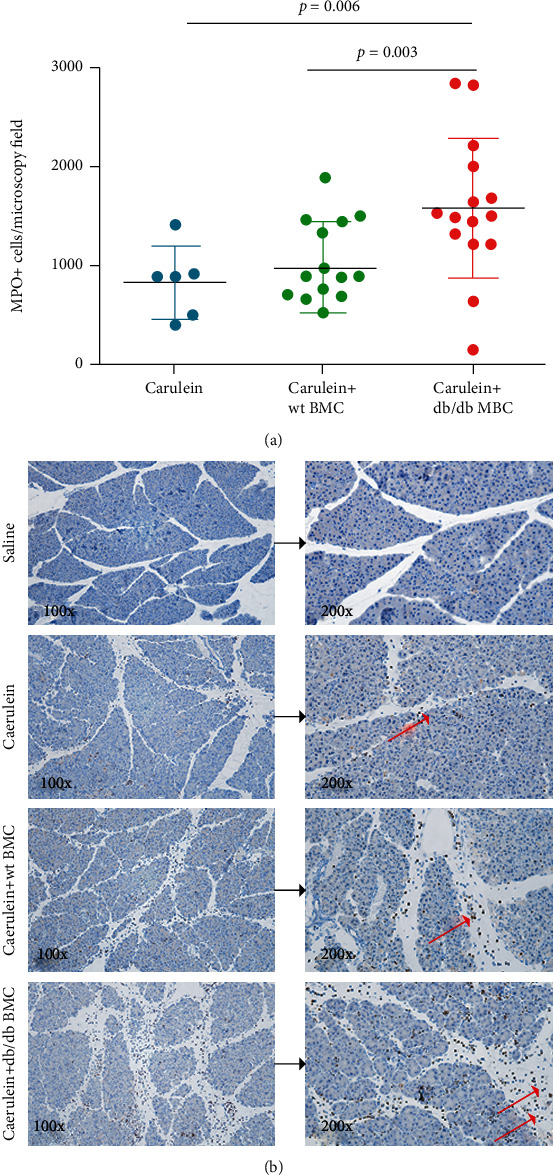
The effect of bone marrow cell injection on the inflammation of AP. Paraffin sections were stained with an antibody against MPO. (a) The number of MPO+ cells per microscopy field was numerated by ImageJ. (b) Representative pictures of MPO-stained sections in wt mice that received saline injection and caerulein injection without or with bone marrow cell injection. AP: acute pancreatitis; MPO: myeloperoxidase; wt: wild type.

Conventionally, circulating neutrophils are the first cells homing to the injured site to participate in inflammation. To study the inflammatory status, the sections were stained with an antibody against MPO, the general marker of neutrophils. Similar to the severity score, the number of MPO+ cells per microscopy field was comparable between the no injection and wt BMC injection groups (834.3 ± 360.0 cells/field vs. 984.9 ± 448.8 cells/field, *p* = 0.47). Nevertheless, the number of MPO+ cells was 1.9- and 1.6-folds higher in mice that received db/db BMC injection compared with wt BMC injection and no BMC injection, respectively (*p* ≤ 0.02 for both) (Figure 4(a)). Moreover, MPO+ cells were 1.6-folds greater in mice injected with db/db BMC than those with wt BMC, indicating increased BMC homing to the injured pancreas (1576.0 ± 703.8 cells/field vs. 984.9 ± 448.8 cells/field, *p* = 0.01). Representative MPO-stained sections of each group are shown in Figure 4(b).

**Figure 5:**
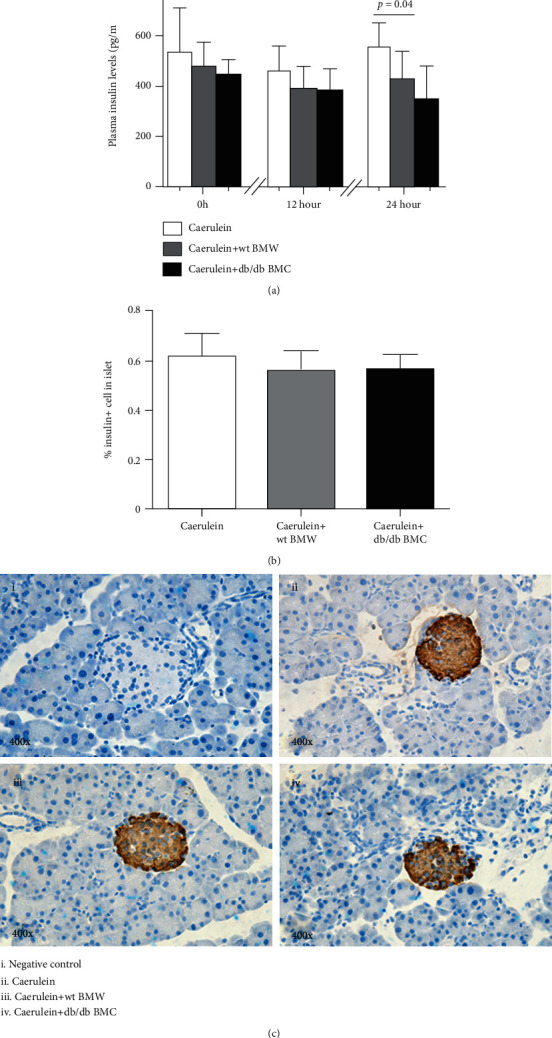
The impact of cell injection on pancreatic *β* cells. (a) Plasma insulin levels at baseline, 12 hours, and 24 hours after the first caerulein injection. *n* = 6-9. (b, c) Pancreas sections were stained with an antibody against insulin, and insulin+ cells in islets were analyzed. *n* = 5 for each group.

By ELISA, plasma insulin levels were reduced in the recipients that received wt BMC and db/db BMC at sacrifice compared with those without cell injection (Figure 5(a)). Nonetheless, when pancreas sections were stained with an antibody against insulin, the percentage of *β* cells in islets was comparable among the three groups (*p* = 0.48) (Figure 5(b)). Representative morphology of insulin-stained islets is shown in Figure 5(c).

Taken together, BMC of diabetic db/db mice triggered more inflammation and acinar cell damage in mice with AP. These data suggest that diabetic BMC were different from wt BMC with inherited inflammatory nature.

### 3.5. Inflammatory Profile of Diabetic db/db BMC

**Figure 6:**
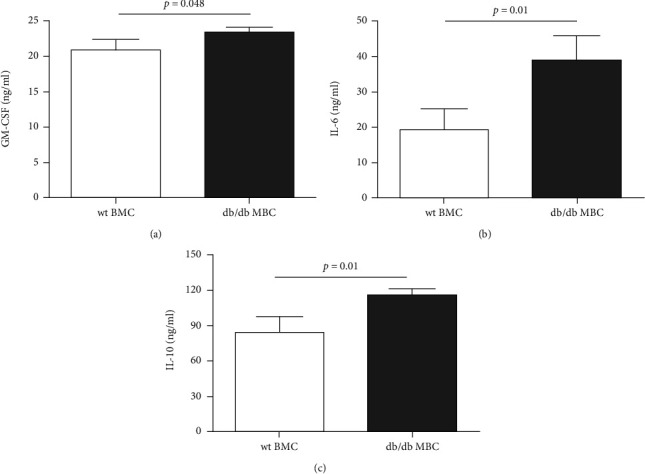
Cytokine production of GM-CSF (a), IL-6 (b), and IL-10 (c) in bone marrow cells isolated from wt and db/db mice at the age of 16 weeks. *n* = 3-4. wt: wild type; BMC: bone marrow cells.

Finally, proteins were extracted from freshly isolated BMC to measure proinflammatory and anti-inflammatory cytokine and chemokine production. Quantified by the Bio-Plex Pro signaling assay, the intracellular levels of GM-CSF, IL-6, and IL-10 were all higher in db/db BMC compared with wt BMC (Figures 6(a)–6(c)). The cytokine and chemokine profiles of wt and db/db BMC are listed in Table 2.

## 4. Discussion

The main findings of the study include the following: (1) at the age of 16 weeks, diabetic db/db mice developed hyperglycemia and an increased amount of granulocytes and monocytes in the peripheral blood; (2) BMC of db/db mice produced more GM-CSF, IL-6, and IL-10 than that of wt controls; and (3) compared with no cell injection, injection of BMC isolated from db/db mice accelerated inflammation and acinar cell damage in mice with AP whereas injection of wt BMC did not aggravate disease progression.

AP is the leading cause of hospital admission in the United States and other countries, resulting in organ failure and mortality [16–18]. Gallstones, alcohol abuse, smoking, and hypertriglyceridemia are the well-known risk factors of AP [16–18]. From the pathological view, AP comprises two waves of inflammation. The first wave initiates from injured acinar cells that secrete inflammatory cytokines to recruit circulating granulocytes and monocytes homing to the lesion site. The second wave comes from infiltrating granulocytes and monocytes that elevate proinflammatory cytokine and chemokine production. Except for inflammatory cytokine secretion and oxidative stress induction, neutrophils release their DNA, histone proteins, high mobility group box 1, and granule components into the pancreas to form neutrophil extracellular traps (NETs), which facilitate inflammation by duct occlusion and trypsin activation [14, 19]. In parallel, NF-*κ*B and STAT3 pathways become activated in monocytes. Ultimately, the inflammatory cascades propagate, leading to systemic inflammation [19].

Recently, a growing body of evidence has indicated that diabetes not only exposes higher risk but also promotes more severe AP than nondiabetic ones [4]. From the mechanism insight, hereby, we demonstrated that BMC of db/db mice had distinct inflammatory cytokine profiles from BMC isolated from wt controls (Figure 5). This partially explains why diabetic BMC injection contributed substantially to the inflammation and acinar cell damage in acute pancreatitis. Among the cytokines that were differentially expressed in wt and diabetic BMC, IL-6 was the major one involved in AP. Via the IL-6 receptor expressed on acinar cells, IL-6/IL-6R activates signal transducers and activators of transcription 3 (STAT3) for acinar cell destruction [20]. The result of a meta-analysis comprising mild AP (*n* = 896), severe AP (*n* = 700), and healthy controls confirmed the positive correlation of plasma IL-6 levels and disease severity [21].

In the study, IL-10 production was increased in diabetic BMC when compared with wt BMC (Figure 5(b)). In line with our findings, Chen et al. reported that serum levels of IL-10 could discriminate the severe type from mild ones in AP [22]. IL-10 is produced by activated T cells such as Treg, Th17, and Th1 cells and macrophages stimulated by bacterial lipopolysaccharides and catecholamines or clearance of apoptotic cells [23]. Through binding to IL-10R1, Janus kinases Jak1 and Tyk2 are activated, resulting in STAT3 phosphorylation and nuclear translocation for target gene transcription. The anti-inflammatory property of IL-10 comes from the inhibition of inflammatory cytokine release and antigen presentation in monocytes and macrophages [23]. Intriguingly, IL-10 derived from plasma cells triggers myeloid lineage differentiation when cocultured in vitro [24]. Thus, how IL-10 participates in inflammation of AP needs future investigation.

Serum lipase and amylase are conventional markers to diagnose AP. Quantified by ELISA, the elevated lipase activity sustained much longer than amylase following the induction of AP by caerulein injection (Figure 2). The inconsistency between serum amylase activity and histological changes in the pancreas was noticed, which could be due to the different half-life and metabolism of these enzymes [25].

The limitations of the study are as follows. First, BMC injection was performed to evaluate the impact of diabetes on AP induced by caerulein injection. In this simplified model, we could not assess the global impact of diabetes such as hyperglycemia on disease progression. Second, we did not evaluate the migratory capacity of injected BMC in vivo. In the study, the recipient mice with induced AP received db/db BMC, wt BMC, or no BMC injection. By MPO staining, the number of MPO+ cells was more pronounced in the recipients that received db/db BMC than wt BMC, implying the increased BMC homing to the lesion site. Whether and how IMP stimulated BMC migration need future investigation. And third, we did not dissect the effect of lymphocytes and macrophages on the progression of AP. By staining with the anti-CD3 or anti-CD20 antibody, T- and B-lymphocytes were detected more in the interstitium than in the acini of patients with AP [26]. By contrast, as immune mediators, certain subtypes of lymphocytes could have a detrimental effect on secondary infection in AP [27]. Similarly, M1 and M2 macrophages have distinct properties in disease progression. M1 macrophages are proinflammatory and secrete inflammatory cytokines and chemokines in AP whereas M2 macrophages could facilitate stellate cells for fibrotic formation in chronic pancreatitis [28]. It would be of great interest to explore how these cells orchestrate the inflammatory progression of pancreatitis.

Taken together, following diabetes progression, the number of myeloid cells increases whereas lymphocytes decrease. BMC of diabetic db/db mice had different features from that of wt ones in that they produce more proinflammatory cytokines such as IL-6. In response to acinar cell injury, diabetic BMC aggravated the inflammation cascade and acinar cell injury, leading to AP progression.

## Figures and Tables

**Table 1:** General characteristics of wild-type and db/db mice at the age of 16 weeks.

	Wild-type mice	db/db mice	*p* value
Number	5-11	10-12	
Total cholesterol (mmol/L)	1.95 ± 0.24	4.24 ± 0.74	0.0001
Total triglyceride (mmol/L)	0.61 ± 0.13	1.55 ± 0.82	0.026
Plasma glucose (mmol/L)	11.02 ± 1.79	34.11 ± 0.82	0.0001
White blood cells (×10^9^/L)	3.48 ± 0.92	2.09 ± 0.65	0.0005
Lymphocytes (×10^9^/L)	2.72 ± 0.71	1.20 ± 0.40	0.0001
Monocytes (×10^9^/L)	0.11 ± 0.04	0.22 ± 0.05	0.0003
Granulocytes (×10^9^/L)	0.47 ± 0.10	0.88 ± 0.21	0.0001
Lymphocytes (%)	78.14 ± 4.68	50.69 ± 6.20	0.0001
Monocytes (%)	4.57 ± 1.71	9.26 ± 1.12	0.0001
Granulocytes (%)	17.27 ± 3.38	40.21 ± 5.88	0.0001
Red blood cells (×10^12^/L)	2.92 ± 0.28	3.84 ± 0.23	0.0001
Hemoglobin (gram)	43.03 ± 4.55	57.90 ± 22.03	0.04
Platelets (×10^9^/L)	785.80 ± 123.59	530.96 ± 129.93	0.0001
Mean platelet volume (fL)	12.67 ± 0.50	11.7 ± 1.69	0.07

Data were expressed as mean ± SD.

**Table 2:** Summary of cytokines produced by bone marrow cells from wild-type and db/db mice at the age of 16 weeks.

Cytokines	Wild-type mice	db/db mice	*p* value
Number	4	4	
GM-CSF (pg/mL)	20.8 ± 1.5	24.1 ± 0.6	0.048
IFN-*γ* (pg/mL)	5.98 ± 4.25	8.32 ± 1.09	0.13
IL-1*α* (pg/mL)	27.7 ± 3.1	30.9 ± 1.4	0.59
IL-1*β* (pg/mL)	45.0 ± 20.5	54.9 ± 8.7	0.44
IL-2 (pg/mL)^∗^	4.18 ± 1.67	3.61 ± 0.70	0.60
IL-4 (pg/mL)	4.19 ± 0.99	4.46 ± 0.80	0.80
IL-5 (pg/mL)	4.69 ± 0.54	4.84 ± 0.90	0.90
IL-6 (pg/mL)	19.3 ± 5.9	37.4 ± 7.0	0.01
IL-7 (pg/mL)	8.72 ± 2.37	12.25 ± 1.91	0.063
IL-10 (pg/mL)	84.2 ± 13.1	108.5 ± 11.6	0.03
IL-12 (p70) (pg/mL)	38.3 ± 3.3	42.6 ± 5.6	0.12
KC (pg/mL)	605.7 ± 918.5	112.0 ± 30.9	0.27
MCP-1 (pg/mL)	95.6 ± 60.4	71.6 ± 18.8	0.41
TNF-*α* (pg/mL)	7.22 ± 3.82	6.63 ± 0.61	0.75

GM-CSF: granulocyte-macrophage colony-stimulating factor; IFN-*γ*: interferon-gamma; IL-1*α*: interleukin-1alpha; IL-1*β*: interleukin-1beta; IL-2: interleukin-2; IL-4: interleukin-4; IL-5: interleukin-5; IL-6: interleukin-6; IL-7: interleukin-7; IL-10: interleukin-10; IL-12 (p70): interleukin-12; IL-13: interleukin-13; IL-17A: interleukin-17A; KC: keratinocyte chemokine; LIX: lipopolysaccharide-induced CXC chemokine; MCP-1: monocyte chemotactic protein-1; MIP-2: macrophage inflammatory protein-2; TNF-*α*: tumor necrosis factor-alpha. In the assay, the values of IL-13, IL-17 (IL-17A), LIX, and MIP-2 were under the detection limit in both groups. Data were expressed as mean ± SD.

## Data Availability

The data in the study are available from the corresponding author upon request.
