# Correction to “Diabetic Bone Marrow Cell Injection Accelerated Acute Pancreatitis Progression”

**DOI:** 10.1155/jimr/9856497

**Published:** 2026-05-10

X‐M. Luo, C. Yan, Y‐J. Zhang, et al., “Diabetic Bone Marrow Cell Injection Accelerated Acute Pancreatitis Progression,” *Journal of Immunology Research*, 2021, https://doi.org/10.1155/2021/5123823.

In the article, errors were introduced in Figure 3f during the production process. Specifically:

• In the Caerulein + wt BMC group, the 200x image was misplaced as the Caerulein + db/db BMC 100x image.
• The Caerulein + db/db BMC 200x image was incorrectly duplicated in the Caerulein + db/db BMC 200x panel.

The correct Figure 3 is below:

**Figure 3:**
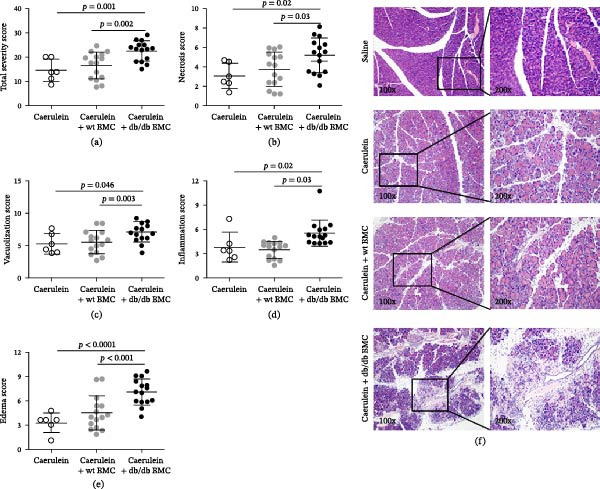
The effect of bone marrow cell injection on AP progression. The severity was assessed on H&E–stained sections in the aspects of acinar cell necrosis and vacuolization, inflammatory cell infiltration, and edema of the pancreas. The degree of severity was scored from 1 to 10. (a) Total severity score was the sum of necrosis (b), vacuolization (c), inflammation (d), and edema (e). *n* = 6–15. AP, acute pancreatitis.

We apologize for this error.

